# Crystal structure of poly[di­aqua­bis­(μ_5_-benzene-1,3-di­carboxyl­ato)(*N*,*N*-di­methyl­formamide)­cadmium(II)disodium(I)]

**DOI:** 10.1107/S2056989017013871

**Published:** 2017-10-03

**Authors:** Matimon Sangsawang, Kittipong Chainok, Nanthawat Wannarit

**Affiliations:** a Department of Chemistry, Faculty of Science and Technology, Thammasat University, Khlong Laung, Pathumthani 12121, Thailand; b Materials and Textile Technology, Faculty of Science and Technology, Thammasat University, Khlong Laung, Pathumthani 12121, Thailand

**Keywords:** crystal structure, Cd^II^–Na^I^ bimetallic, three-dimensional framework

## Abstract

The title compound, [CdNa_2_(C_8_H_4_O_4_)_2_(C_3_H_7_NO)(H_2_O)_2_]_*n*_ or [CdNa_2_(1,3-bdc)_2_(DMF)(H_2_O)_2_]_*n*_, is a new Cd^II^–Na^I^ heterobimetallic coordination polymer. The asymmetric unit consists of one Cd^II^ atom, two Na^I^ atoms, two 1,3-bdc ligands, two coordinated water mol­ecules and one coordinated DMF mol­ecule. The Cd^II^ atom exhibits a seven-coordinate geometry, while the Na^I^ atoms can be considered to be penta­coordinate. The metal ions and their symmetry-related equivalents are connected *via* chelating–bridging carboxyl­ate groups of the 1,3-bdc ligands to generate a three-dimensional framework. In the crystal, there are classical O—H⋯O hydrogen bonds involving the coordinated water mol­ecules and the 1,3-bdc carboxyl­ate groups and π–π stacking between the benzene rings of the 1,3-bdc ligands present within the frameworks.

## Abstract 2

A novel three-dimensional bimetallic Cd^II^–Na^I^ metal–organic framework has been synthesized and the X-ray structure has been reported.

## Chemical context   

Porous coordination polymers or metal–organic frameworks (MOFs) constructed from *d* ^10^ transition metals and benzene polycarboxyl­ate bridging ligands have been widely studied (Yaghi *et al.*, 1999 ▸; Lin *et al.*, 2008 ▸; Seco *et al.*, 2017 ▸) due to the varieties of coordination framework topologies and also potential applications in gas adsorption (Suh *et al.*, 2012 ▸), photoluminescence (Wang *et al.*, 2012 ▸) and photocatalysis (Wu *et al.*, 2017 ▸). Among the most common ligands in this class, the rigid and planar backbone of benzene di­carboxyl­ates such as benzene-1,3-di­carb­oxy­lic acid (1,3-H_2_bdc) and benzene-1,4-di­carb­oxy­lic acid (1,4-H_2_bdc) are widely employed in the construction of these solids owing to their rich coordination modes. Studies incorporating alkaline metal ions into *d* ^10^-MOFs with one type of bridging ligand to construct novel heterobimetallic *d* ^10^-alkaline metal ion MOFs have been undertaken (Lin *et al.*, 2010*a* ▸,*b* ▸). The alkali metal ions could provide an unpredictable coordination number and pH-dependent self-assembly in the construction of coordination frameworks with various types of topology and dimensionality (Borah *et al.*, 2011 ▸; Chen *et al.*, 2011 ▸). However, the members of three-dimensional coordination framework heterobi­metallic Zn^II^ or Cd^II^ /Na^I^ MOFs with benzene­polycarboxyl­ate ligands are still limited; previous reports include [ZnNa(1,2,4-btc)] where 1,2,4-btc = benzene-1,2,4-tri­carboxyl­ate (Wang *et al.*, 2004 ▸), [Zn_2_Na_2_(1,4-bdc)_3_·(DMF)_2_·(*m*-H_2_O)_2_] where 1,4-bdcH_2_ = benzene-1,4-di­carb­oxy­lic acid (Xu *et al.*, 2004 ▸), {[CdNa(1,3-bdc)_2_]·[NH_2_(CH_3_)_2_]} where 1,3-bdcH_2_ = benzene-1,3-di­carb­oxy­lic acid (Che *et al.*, 2007 ▸), [CdNa(OH-1,3-bdc)_2_(H_2_O)_2_]·2H_2_O where OH-1,3-bdcH_2_ = 5-hy­droxy­benzene-1,3-di­carb­oxy­lic acid (Du *et al.*, 2013 ▸) and [Cd_8_Na(ntc)_6_(H_2_O)_8_] where ntcH_3_ = 5-nitro­benzene-1,2,3-tri­carb­oxy­lic acid (Yang *et al.*, 2014 ▸). With the aim of searching for new members of this heterobimetallic MOFs system containing benzene-1,3-di­carb­oxy­lic acid (1,3-bdcH_2_), we explored mixed sources of Zn^II^/Cd^II^–Na^I^ with this ligand. The expected products are prepared by using a direct synthetic method, mixing metal nitrate salts, 1,3-bdcH_2_ and NaOH (mole ratio 1:1:2) in water, methanol and DMF solvents. However, only the Cd^II^–Na^I^ MOF product has been successfully synthesized. As part of our ongoing studies on this complex, we describe here the synthesis and crystal structure of a novel three-dimensional heterobimetallic Cd^II^–Na^I^ MOF, [CdNa_2_(1,3-bdc)_2_(DMF)(H_2_O)_2_]_*n*_ (**I**).
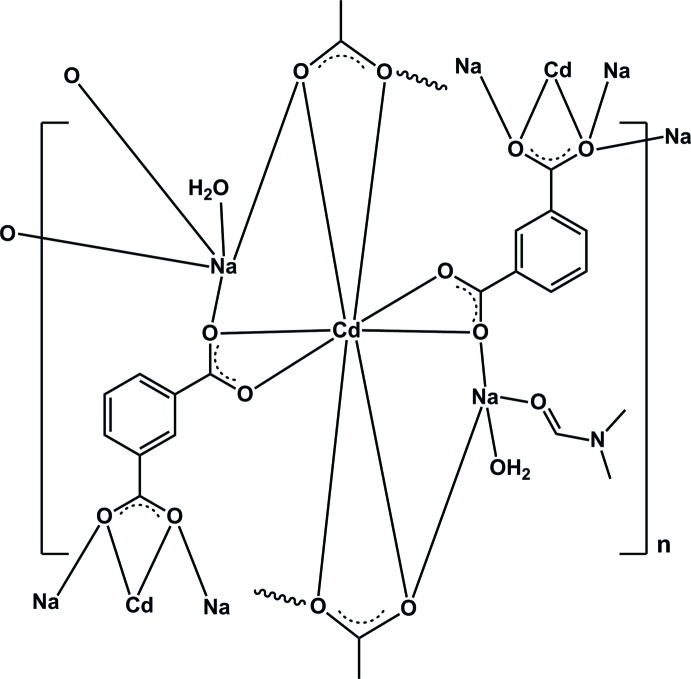


## Structural commentary   

**Figure 1:**
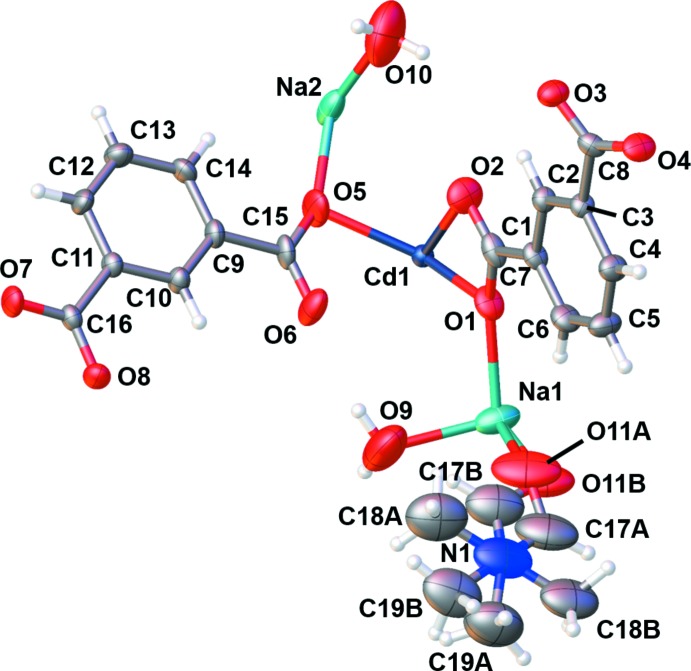
Asymmetric unit of (**I**) with the atomic-numbering scheme. Displacement ellipsoids are drawn at the 50% probability level.

**Figure 2:**
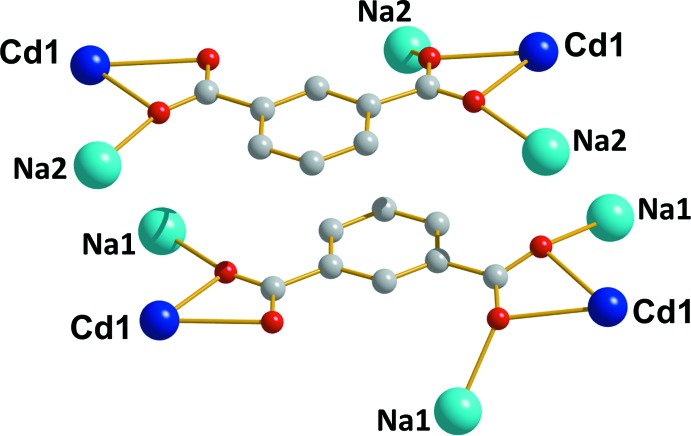
Coordination mode of the μ_5_-1,3-bdc bridging ligands found in (**I**). All hydrogen atoms are omitted for clarity.

**Figure 3:**
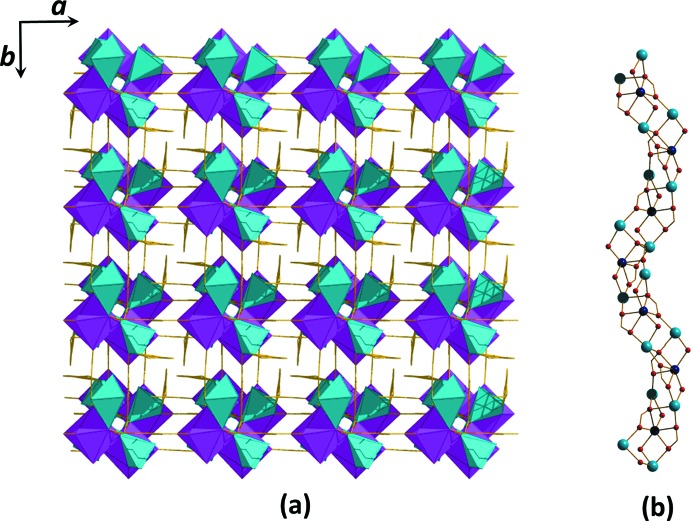
Perspective view along the crystallographic *c* axis of (*a*) the three-dimensional framework of (**I**) (the coordination polyhedra for Cd^II^ and Na^I^ are pink and green, respectively) and (*b*) helical chain-like structure of the Cd—Na clusters (dark blue = Cd, blue = Na and red = O).

**Figure 4:**
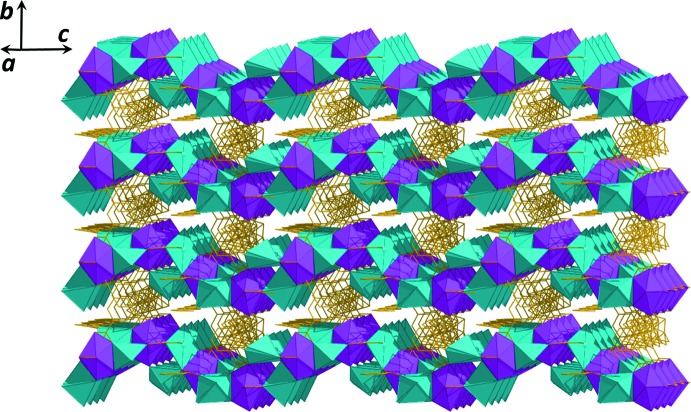
Perspective view of the three-dimensional framework of (**I**) (the coordination polyhedra for Cd^II^ and Na^I^ are pink and green, respectively). All hydrogen atoms are omitted for clarity.

The title compound (**I**) crystallizes in the tetra­gonal crystal system with polar *P*4_3_ space group. The asymmetric unit of (**I**) consists of one Cd^II^ ion, two crystallographically independent Na(I) ions, two 1,3-bdc ligands, two coordinated water mol­ecules and one DMF mol­ecules, as shown in Fig. 1 ▸. Each Cd^II^ ion is coordinated by seven carboxyl­ate oxygen atoms from four different 1,3-bdc ligands with the Cd—O bond distances range between 2.301 (3) and 2.555 (3) Å (Table 1 ▸). The Na1 ion is surrounded by three carboxyl­ate oxygen atoms of three different 1,3-bdc ligands, one oxygen atom from a water mol­ecule, and one DMF mol­ecule with the Na—O bond distances ranging between 2.304 (7) and 2.498 (11) Å, while the Na2 ion adopts a five-coordinate [4 + 1] coordination with four oxygen atoms from three different 1,3-bdc ligands and one oxygen atom from a water mol­ecule. The Na—O bond distances are in the range 2.275 (5) to 2.354 (8) Å. Fig. 2 ▸ shows the coordination modes of the 1,3-bdc ligand in compound (**I**). The 1,3-bdc mol­ecule is fully deprotonated and coordinated to the Cd^II^ and Na^I^ ions in a μ_5_-coordination mode, creating a one-dimensional heterobimetallic chain running parallel to the *c* axis, Fig. 3 ▸. Adjacent chains are further connected through the 1,3-bdc ligands in the *a*- and *b*-axis directions, generating a three-dimensional framework structure as shown in Fig. 4 ▸. The coordinated water and DMF mol­ecules adopt a monodentate coordination mode and serve as a terminal pendant ligand pointing inside the channels.

## Supra­molecular features   

In the crystal of (**I**), classical O—H⋯O hydrogen bonds and aromatic π–π stacking inter­actions are observed and these inter­actions presumably help to stabilize the frameworks. All water mol­ecules are shown to act as O—H⋯O hydrogen-bond donors towards the carboxyl­ate groups of the 1,3-bdc ligands (Table 2 ▸). The π–π stacking inter­actions are between symmetry-related aromatic rings of the 1,3-bdc ligands with a *Cg*1⋯*C*g2^i^ distance of 3.588 (3) Å and a dihedral angle of 3.8 (4)° [*Cg*1 and *Cg*2 are the centroids of the C1–C6 and C9–C14 rings, respectively; symmetry code: (i) –*y*, *x*, *z* – 1/4].

## Database survey   

To the best of our knowledge of structures closely related to (**I**), only the three-dimensional coordination framework {[CdNa(1,3-bdc)_2_]·[NH_2_(CH_3_)_2_]} has been reported (Che *et al.*, 2007 ▸). This compound crystallized in the centrosymmetric space group *C*2/*c*. The Cd^II^ and Na^I^ centers are linked by a 1,3-bdc ligand in a μ_4_-coordination mode. The DMF solvent decomposes under solvothermal synthesis, with the construction of a 3D coordination framework with open channels containing NH_2_(CH_3_)_2_ mol­ecules. In comparison, compound (**I**) contains coordinated H_2_O and DMF mol­ecules projecting into the framework channels. Other related three-dimensional heterobimetallic *d* ^10^–Na^I^ coordination frameworks containing benzene­polycarboxyl­ate ligands have been published, such as [CdNa(OH-1,3-bdc)_2_(H_2_O)_2_]·2H_2_O where OH-1,3-bdcH_2_ = 5-hy­droxy-benzene-1,3-di­carb­oxy­lic acid (Du *et al.*, 2013 ▸), [Zn_2_Na_2_(1,4-bdc)_3_·(DMF)_2_·(*m*-H_2_O)_2_] where 1,4-bdcH_2_ = benzene-1,4-di­carb­oxy­lic acid (Xu *et al.*, 2004 ▸), [ZnNa(1,2,4-btc)] where 1,2,4-btc = 1,2,4-benzene­tri­carboxyl­ate (Wang *et al.*, 2004 ▸), and [Cd_8_Na(ntc)_6_(H_2_O)_8_] where ntcH_3_ = 5-nitro­benzene-1,2,3-tri­carb­oxy­lic acid (Yang *et al.*, 2014 ▸). The three-dimensional coordination framework topologies of these compounds are the result of the construction of different types of metal centers, geometries and carboxyl­ate ligand derivatives. It is found that the carboxyl­ate ligand derivatives in the structure of these related compounds exhibit a μ_4_-coordination mode.

## Synthesis and crystallization   

A mixture solution of 1,3-bdcH_2_ (1.0 mmol) and NaOH (2.0 mmol) in 10 mL of distilled water was slowly dropped to a methano­lic solution (10 ml) of Cd(NO_3_)_2_·4H_2_O (1.0 mmol). The reaction mixture was stirred at 333 K for 30 min and allowed to cool to room temperature and then filtered. The filtrate was allowed to stand to slowly evaporate at ambient temperature. Colorless block-shaped crystals suitable for single crystal X-ray diffraction were obtained after three days (76% yield based on Cd).

## Refinement   

Crystal data, data collection and structure refinement details are summarized in Table 3 ▸. All hydrogen atoms except those of water mol­ecules were generated geometrically and refined isotropically using a riding model, with C—H = 0.93 Å and *U*_iso_(H) = 1.2*U*_eq_(C). The coordinated DMF mol­ecule was found to be disordered with two sets of sites with a refined occupancy ratio of 0.382 (10) and 0.618 (10).

## Supplementary Material

Crystal structure: contains datablock(s) I. DOI: 10.1107/S2056989017013871/pj2046sup1.cif

Structure factors: contains datablock(s) I. DOI: 10.1107/S2056989017013871/pj2046Isup2.hkl

CCDC reference: 1576543

Additional supporting information:  crystallographic information; 3D view; checkCIF report

## Figures and Tables

**Table 1:** Selected geometric parameters (Å, °)

Cd1—O1	2.301 (3)	Na1—O4^i^	2.441 (5)
Cd1—O2	2.555 (3)	Na1—O9	2.304 (7)
Cd1—O3^i^	2.496 (3)	Na1—O11*B*	2.498 (11)
Cd1—O4^i^	2.385 (3)	Na1—O11*A*	2.475 (18)
Cd1—O5	2.284 (4)	Na2—O4^iv^	2.655 (5)
Cd1—O7^ii^	2.396 (3)	Na2—O5	2.277 (5)
Cd1—O8^ii^	2.472 (3)	Na2—O7^ii^	2.282 (4)
Na1—O1	2.368 (5)	Na2—O8^v^	2.275 (5)
Na1—O3^iii^	2.339 (5)	Na2—O10	2.354 (8)
			
O1—Cd1—O2	53.12 (15)	O1—Na1—O4^i^	77.84 (17)
O1—Cd1—O3^i^	131.59 (15)	O1—Na1—O11*B*	104.1 (3)
O1—Cd1—O4^i^	80.31 (12)	O3^iii^—Na1—O1	151.1 (2)
O1—Cd1—O7^ii^	125.91 (12)	O3^iii^—Na1—O4^i^	94.59 (15)
O1—Cd1—O8^ii^	92.04 (13)	O3^iii^—Na1—O11*B*	82.9 (3)
O3^i^—Cd1—O2	173.00 (16)	O4^i^—Na1—O11*B*	177.4 (3)
O4^i^—Cd1—O2	132.60 (15)	O9—Na1—O1	95.8 (2)
O4^i^—Cd1—O3^i^	53.37 (13)	O9—Na1—O3^iii^	112.0 (2)
O4^i^—Cd1—O7^ii^	122.40 (12)	O9—Na1—O4^i^	88.3 (2)
O4^i^—Cd1—O8^ii^	78.81 (13)	O9—Na1—O11*B*	93.2 (4)
O5—Cd1—O1	125.67 (14)	O5—Na2—O4^iv^	95.45 (19)
O5—Cd1—O2	90.36 (16)	O5—Na2—O7^ii^	83.03 (18)
O5—Cd1—O3^i^	82.65 (15)	O5—Na2—O10	104.5 (2)
O5—Cd1—O4^i^	128.83 (14)	O7^ii^—Na2—O4^iv^	94.98 (14)
O5—Cd1—O7^ii^	80.41 (12)	O7^ii^—Na2—O10	80.5 (2)
O5—Cd1—O8^ii^	133.24 (16)	O8^v^—Na2—O4^iv^	77.02 (13)
O7^ii^—Cd1—O2	85.03 (15)	O8^v^—Na2—O5	110.18 (16)
O7^ii^—Cd1—O3^i^	94.01 (14)	O8^v^—Na2—O7^ii^	164.93 (18)
O7^ii^—Cd1—O8^ii^	53.55 (14)	O8^v^—Na2—O10	102.3 (2)
O8^ii^—Cd1—O2	93.07 (11)	O10—Na2—O4^iv^	158.8 (2)
O8^ii^—Cd1—O3^i^	91.92 (10)		

Symmetry codes: (i) 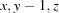; (ii) 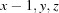; (iii) 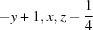; (iv) 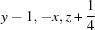; (v) 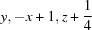.

**Table 2:** Hydrogen-bond geometry (Å, °)

*D*—H⋯*A*	*D*—H	H⋯*A*	*D*⋯*A*	*D*—H⋯*A*
O9—H9*B*⋯O6	0.90	2.22	3.074 (8)	159
O10—H10*B*⋯O6^v^	0.86	2.29	3.073 (8)	152

Symmetry code: (v) 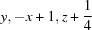.

**Table 3:** Experimental details

Crystal data	
Chemical formula	[CdNa_2_(C_8_H_4_O_4_)_2_(C_3_H_7_NO)(H_2_O)_2_]
*M*_r_	595.73
Crystal system, space group	Tetragonal, *P*4_3_
Temperature (K)	296
*a*, *c* (Å)	10.1437 (8), 21.4664 (15)
*V* (Å^3^)	2208.8 (4)
*Z*	4
Radiation type	Mo *K*α
μ (mm^−1^)	1.09
Crystal size (mm)	0.35 × 0.21 × 0.16

Data collection	
Diffractometer	Bruker APEXII D8 QUEST CMOS
Absorption correction	Multi-scan (*SADABS*, Bruker, 2013 ▸)
*T*_min_, *T*_max_	0.647, 0.704
No. of measured, independent and observed [*I* > 2σ(*I*)] reflections	56814, 5708, 5301
*R*_int_	0.074
(sin θ/λ)_max_ (Å^−1^)	0.676

Refinement	
*R*[*F* ^2^ > 2σ(*F* ^2^)], *wR*(*F* ^2^), *S*	0.028, 0.068, 1.03
No. of reflections	5708
No. of parameters	351
No. of restraints	160
H-atom treatment	H-atom parameters constrained
Δρ_max_, Δρ_min_ (e Å^−3^)	0.52, −0.45
Absolute structure	Flack *x* determined using 2427 quotients [(*I* ^+^)−(*I* ^−^)]/[(*I* ^+^)+(*I* ^−^)] (Parsons *et al*., 2013 ▸)
Absolute structure parameter	0.081 (13)

Computer programs: *APEX2* and *SAINT* (Bruker, 2013 ▸), *SHELXT* (Sheldrick, 2015*a* ▸), *SHELXL* (Sheldrick, 2015*b* ▸) and *OLEX2* (Dolomanov *et al.*, 2009 ▸).

## supplementary crystallographic information

### Crystal data

**Table:**

[CdNa_2_(C_8_H_4_O_4_)_2_(C_3_H_7_NO)(H_2_O)_2_]	*D*_x_ = 1.791 Mg m^−^^3^
*M**_r_* = 595.73	Mo *K*α radiation, λ = 0.71073 Å
Tetragonal, *P*4_3_	Cell parameters from 9769 reflections
*a* = 10.1437 (8) Å	θ = 2.7–28.3°
*c* = 21.4664 (15) Å	µ = 1.09 mm^−^^1^
*V* = 2208.8 (4) Å^3^	*T* = 296 K
*Z* = 4	Block, colourless
*F*(000) = 1192	0.35 × 0.21 × 0.16 mm

### Data collection

**Table:**

Bruker APEXII D8 QUEST CMOS diffractometer	5301 reflections with *I* > 2σ(*I*)
Detector resolution: 10.5 pixels mm^-1^	*R*_int_ = 0.074
ω and φ scans	θ_max_ = 28.7°, θ_min_ = 2.8°
Absorption correction: multi-scan (SADABS, Bruker, 2013)	*h* = −13→13
*T*_min_ = 0.647, *T*_max_ = 0.704	*k* = −12→13
56814 measured reflections	*l* = −28→29
5708 independent reflections	

### Refinement

**Table:**

Refinement on *F*^2^	H-atom parameters constrained
Least-squares matrix: full	*w* = 1/[σ^2^(*F*_o_^2^) + (0.0375*P*)^2^ + 0.6193*P*] where *P* = (*F*_o_^2^ + 2*F*_c_^2^)/3
*R*[*F*^2^ > 2σ(*F*^2^)] = 0.028	(Δ/σ)_max_ < 0.001
*wR*(*F*^2^) = 0.068	Δρ_max_ = 0.52 e Å^−^^3^
*S* = 1.03	Δρ_min_ = −0.45 e Å^−^^3^
5708 reflections	Extinction correction: SHELXL, Fc^*^=kFc[1+0.001xFc^2^λ^3^/sin(2θ)]^-1/4^
351 parameters	Extinction coefficient: 0.0050 (7)
160 restraints	Absolute structure: Flack *x* determined using 2427 quotients [(*I*^+^)-(*I*^-^)]/[(*I*^+^)+(*I*^-^)] (Parsons et al., 2013)
Hydrogen site location: mixed	Absolute structure parameter: 0.081 (13)

### Special details

**Table:**

Geometry. All esds (except the esd in the dihedral angle between two l.s. planes) are estimated using the full covariance matrix. The cell esds are taken into account individually in the estimation of esds in distances, angles and torsion angles; correlations between esds in cell parameters are only used when they are defined by crystal symmetry. An approximate (isotropic) treatment of cell esds is used for estimating esds involving l.s. planes.

### Fractional atomic coordinates and isotropic or equivalent isotropic displacement parameters (Å^2^)

**Table:**

	*x*	*y*	*z*	*U*_iso_*/*U*_eq_	Occ. (<1)
Cd1	0.08591 (2)	0.23028 (2)	0.49814 (3)	0.02155 (9)	
Na1	0.1091 (4)	0.2329 (2)	0.33008 (17)	0.0575 (9)	
Na2	0.1037 (2)	0.2125 (3)	0.66027 (15)	0.0510 (8)	
O1	0.0894 (3)	0.3769 (3)	0.41640 (17)	0.0357 (8)	
O2	0.0886 (4)	0.4817 (3)	0.5052 (3)	0.0458 (10)	
O3	0.1045 (3)	0.9851 (3)	0.5013 (2)	0.0349 (6)	
O4	0.0482 (4)	1.0819 (3)	0.41384 (15)	0.0354 (8)	
O5	0.2394 (3)	0.2174 (5)	0.57576 (19)	0.0502 (10)	
O6	0.3488 (3)	0.2217 (4)	0.4890 (2)	0.0490 (10)	
O7	0.9433 (3)	0.2411 (4)	0.58696 (16)	0.0337 (7)	
O8	0.8424 (3)	0.2200 (3)	0.4971 (2)	0.0319 (6)	
O9	0.3263 (6)	0.1781 (9)	0.3477 (3)	0.111 (2)	
H9A	0.3381	0.0906	0.3450	0.166*	
H9B	0.3540	0.2025	0.3857	0.166*	
O10	0.0517 (7)	0.4353 (7)	0.6789 (3)	0.118 (2)	
H10A	0.0182	0.4694	0.6461	0.178*	
H10B	0.1218	0.4773	0.6885	0.178*	
C1	0.0879 (4)	0.6114 (4)	0.4125 (2)	0.0251 (8)	
C2	0.0830 (4)	0.7316 (4)	0.4438 (2)	0.0236 (8)	
H2	0.0798	0.7328	0.4871	0.028*	
C3	0.0829 (4)	0.8496 (4)	0.4111 (2)	0.0225 (8)	
C4	0.0860 (5)	0.8478 (4)	0.3463 (2)	0.0312 (10)	
H4	0.0847	0.9267	0.3242	0.037*	
C5	0.0909 (5)	0.7295 (4)	0.3148 (2)	0.0364 (12)	
H5	0.0935	0.7284	0.2715	0.044*	
C6	0.0918 (5)	0.6114 (4)	0.3481 (2)	0.0335 (10)	
H6	0.0951	0.5317	0.3268	0.040*	
C7	0.0881 (4)	0.4829 (4)	0.4476 (3)	0.0314 (10)	
C8	0.0788 (4)	0.9796 (4)	0.4444 (2)	0.0249 (8)	
C9	0.4717 (4)	0.2316 (4)	0.5837 (2)	0.0257 (8)	
C10	0.5928 (4)	0.2363 (4)	0.5534 (2)	0.0245 (9)	
H10	0.5962	0.2377	0.5101	0.029*	
C11	0.7089 (4)	0.2390 (4)	0.58795 (19)	0.0220 (8)	
C12	0.7034 (4)	0.2399 (5)	0.6526 (2)	0.0286 (9)	
H12	0.7809	0.2424	0.6758	0.034*	
C13	0.5834 (4)	0.2370 (4)	0.6825 (2)	0.0327 (10)	
H13	0.5802	0.2389	0.7258	0.039*	
C14	0.4674 (4)	0.2313 (4)	0.6486 (2)	0.0285 (9)	
H14	0.3867	0.2273	0.6691	0.034*	
C15	0.3461 (4)	0.2229 (4)	0.5460 (2)	0.0300 (9)	
C16	0.8396 (4)	0.2330 (4)	0.5549 (2)	0.0227 (8)	
O11B	0.1647 (14)	0.3817 (14)	0.2412 (5)	0.082 (3)	0.618 (10)
N1	0.3469 (8)	0.4165 (5)	0.1823 (3)	0.0859 (17)	
C17B	0.2980 (15)	0.3983 (11)	0.2335 (6)	0.088 (3)	0.618 (10)
H17B	0.3526	0.3957	0.2683	0.106*	0.618 (10)
C18B	0.2609 (14)	0.4209 (11)	0.1244 (7)	0.093 (3)	0.618 (10)
H18A	0.2369	0.3328	0.1127	0.140*	0.618 (10)
H18B	0.3088	0.4617	0.0910	0.140*	0.618 (10)
H18C	0.1828	0.4711	0.1330	0.140*	0.618 (10)
C19B	0.4849 (13)	0.4221 (13)	0.1669 (8)	0.099 (3)	0.618 (10)
H19A	0.5354	0.4346	0.2043	0.149*	0.618 (10)
H19B	0.5005	0.4943	0.1390	0.149*	0.618 (10)
H19C	0.5109	0.3411	0.1473	0.149*	0.618 (10)
C18A	0.439 (2)	0.424 (2)	0.2384 (9)	0.105 (5)	0.382 (10)
H18D	0.3884	0.4164	0.2760	0.157*	0.382 (10)
H18E	0.4851	0.5063	0.2380	0.157*	0.382 (10)
H18F	0.5015	0.3527	0.2364	0.157*	0.382 (10)
C19A	0.433 (3)	0.417 (3)	0.1269 (10)	0.110 (6)	0.382 (10)
H19D	0.4565	0.5063	0.1169	0.165*	0.382 (10)
H19E	0.3865	0.3786	0.0923	0.165*	0.382 (10)
H19F	0.5109	0.3670	0.1353	0.165*	0.382 (10)
C17A	0.2249 (19)	0.401 (2)	0.1936 (11)	0.098 (4)	0.382 (10)
H17A	0.1610	0.3926	0.1628	0.117*	0.382 (10)
O11A	0.198 (3)	0.396 (2)	0.2554 (10)	0.102 (5)	0.382 (10)

### Atomic displacement parameters (Å^2^)

**Table:**

	*U*^11^	*U*^22^	*U*^33^	*U*^12^	*U*^13^	*U*^23^
Cd1	0.02087 (14)	0.02266 (15)	0.02112 (12)	0.00064 (10)	−0.00023 (11)	−0.00002 (11)
Na1	0.096 (2)	0.0428 (14)	0.0337 (16)	−0.0075 (12)	0.0048 (15)	−0.0055 (8)
Na2	0.0289 (10)	0.0944 (19)	0.0298 (14)	−0.0066 (10)	−0.0059 (9)	0.0114 (12)
O1	0.0375 (18)	0.0144 (13)	0.055 (2)	−0.0009 (11)	−0.0018 (15)	0.0020 (13)
O2	0.062 (2)	0.0262 (15)	0.049 (3)	−0.0022 (14)	−0.001 (2)	0.0105 (18)
O3	0.0471 (16)	0.0271 (13)	0.0305 (15)	0.0009 (12)	−0.0002 (19)	−0.0023 (17)
O4	0.055 (2)	0.0144 (13)	0.0365 (18)	0.0024 (13)	0.0009 (15)	0.0005 (12)
O5	0.0156 (15)	0.083 (3)	0.052 (2)	−0.0012 (16)	−0.0008 (14)	0.005 (2)
O6	0.0319 (17)	0.079 (3)	0.036 (3)	−0.0123 (16)	−0.0103 (17)	0.007 (2)
O7	0.0159 (13)	0.048 (2)	0.0369 (17)	−0.0016 (13)	−0.0045 (12)	0.0016 (15)
O8	0.0263 (13)	0.0429 (15)	0.0265 (13)	0.0009 (11)	0.0044 (17)	0.0006 (18)
O9	0.087 (4)	0.193 (8)	0.052 (3)	0.004 (5)	−0.013 (3)	0.003 (4)
O10	0.113 (5)	0.122 (5)	0.120 (6)	−0.002 (4)	−0.047 (4)	0.049 (4)
C1	0.025 (2)	0.0149 (17)	0.035 (2)	−0.0004 (15)	0.0015 (16)	0.0031 (16)
C2	0.025 (2)	0.0183 (19)	0.027 (2)	−0.0009 (15)	−0.0013 (15)	0.0021 (14)
C3	0.026 (2)	0.0151 (18)	0.026 (2)	−0.0021 (14)	−0.0009 (16)	0.0006 (15)
C4	0.041 (3)	0.022 (2)	0.030 (2)	0.0029 (18)	0.0003 (19)	0.0024 (17)
C5	0.055 (3)	0.028 (2)	0.027 (2)	0.002 (2)	0.0015 (19)	−0.0009 (16)
C6	0.038 (3)	0.020 (2)	0.042 (3)	0.0019 (18)	0.001 (2)	−0.0050 (18)
C7	0.022 (2)	0.0180 (19)	0.054 (3)	−0.0016 (15)	−0.0008 (19)	0.0063 (19)
C8	0.0244 (19)	0.0190 (19)	0.031 (2)	−0.0021 (15)	0.0077 (16)	−0.0011 (16)
C9	0.0167 (18)	0.027 (2)	0.033 (2)	−0.0001 (15)	−0.0045 (16)	0.0025 (17)
C10	0.0184 (19)	0.030 (2)	0.026 (2)	−0.0022 (16)	−0.0008 (14)	0.0035 (15)
C11	0.0165 (17)	0.0230 (19)	0.0265 (19)	−0.0025 (14)	−0.0009 (15)	0.0003 (15)
C12	0.023 (2)	0.039 (2)	0.024 (2)	−0.0001 (17)	−0.0057 (16)	−0.0027 (18)
C13	0.032 (2)	0.041 (3)	0.024 (2)	−0.002 (2)	0.0034 (16)	−0.0011 (17)
C14	0.019 (2)	0.034 (2)	0.032 (2)	0.0019 (17)	0.0061 (16)	−0.0008 (18)
C15	0.0143 (18)	0.029 (2)	0.046 (3)	−0.0017 (15)	−0.0069 (18)	0.0079 (19)
C16	0.0159 (17)	0.0210 (18)	0.031 (2)	−0.0006 (14)	0.0001 (15)	0.0015 (15)
O11B	0.125 (6)	0.051 (6)	0.068 (6)	−0.015 (4)	0.025 (4)	−0.027 (5)
N1	0.135 (5)	0.030 (2)	0.093 (3)	0.000 (3)	0.035 (3)	−0.001 (2)
C17B	0.127 (6)	0.047 (5)	0.091 (4)	−0.012 (4)	0.028 (3)	−0.004 (3)
C18B	0.143 (6)	0.041 (6)	0.095 (5)	0.012 (5)	0.029 (4)	−0.008 (4)
C19B	0.135 (5)	0.048 (6)	0.115 (7)	0.001 (4)	0.036 (4)	0.017 (6)
C18A	0.144 (7)	0.073 (12)	0.098 (5)	−0.018 (7)	0.030 (5)	0.008 (6)
C19A	0.136 (8)	0.099 (18)	0.095 (5)	0.006 (9)	0.035 (5)	0.005 (7)
C17A	0.137 (5)	0.057 (10)	0.100 (6)	−0.008 (4)	0.040 (4)	−0.029 (6)
O11A	0.155 (10)	0.052 (9)	0.100 (6)	−0.039 (9)	0.046 (6)	−0.037 (7)

### Geometric parameters (Å, º)

**Table:**

Cd1—O1	2.301 (3)	C1—C7	1.505 (6)
Cd1—O2	2.555 (3)	C2—H2	0.9300
Cd1—O3^i^	2.496 (3)	C2—C3	1.388 (5)
Cd1—O4^i^	2.385 (3)	C3—C4	1.391 (6)
Cd1—O5	2.284 (4)	C3—C8	1.501 (5)
Cd1—O7^ii^	2.396 (3)	C4—H4	0.9300
Cd1—O8^ii^	2.472 (3)	C4—C5	1.379 (6)
Na1—Na2^iii^	3.914 (6)	C5—H5	0.9300
Na1—O1	2.368 (5)	C5—C6	1.395 (7)
Na1—O3^iv^	2.339 (5)	C6—H6	0.9300
Na1—O4^i^	2.441 (5)	C9—C10	1.390 (6)
Na1—O9	2.304 (7)	C9—C14	1.395 (6)
Na1—O11B	2.498 (11)	C9—C15	1.511 (6)
Na1—O11A	2.475 (18)	C10—H10	0.9300
Na2—Cd1^v^	3.791 (3)	C10—C11	1.392 (5)
Na2—Na1^v^	3.914 (6)	C11—C12	1.390 (6)
Na2—O4^vi^	2.655 (5)	C11—C16	1.505 (5)
Na2—O5	2.277 (5)	C12—H12	0.9300
Na2—O7^ii^	2.282 (4)	C12—C13	1.376 (6)
Na2—O8^vii^	2.275 (5)	C13—H13	0.9300
Na2—O10	2.354 (8)	C13—C14	1.385 (6)
O1—C7	1.266 (6)	C14—H14	0.9300
O2—C7	1.237 (8)	O11B—C17B	1.373 (18)
O3—Cd1^viii^	2.495 (3)	N1—C17B	1.221 (13)
O3—Na1^vii^	2.339 (5)	N1—C18B	1.517 (15)
O3—C8	1.250 (6)	N1—C19B	1.439 (14)
O4—Cd1^viii^	2.385 (3)	N1—C18A	1.526 (18)
O4—Na1^viii^	2.442 (5)	N1—C19A	1.473 (17)
O4—Na2^ix^	2.655 (5)	N1—C17A	1.271 (19)
O4—C8	1.266 (5)	C17B—H17B	0.9300
O5—C15	1.259 (6)	C18B—H18A	0.9600
O6—C15	1.224 (7)	C18B—H18B	0.9600
O7—Cd1^x^	2.396 (3)	C18B—H18C	0.9600
O7—Na2^x^	2.282 (4)	C19B—H19A	0.9600
O7—C16	1.259 (5)	C19B—H19B	0.9600
O8—Cd1^x^	2.472 (3)	C19B—H19C	0.9600
O8—Na2^iv^	2.275 (5)	C18A—H18D	0.9600
O8—C16	1.248 (6)	C18A—H18E	0.9600
O9—H9A	0.8971	C18A—H18F	0.9600
O9—H9B	0.8991	C19A—H19D	0.9600
O10—H10A	0.8544	C19A—H19E	0.9600
O10—H10B	0.8548	C19A—H19F	0.9600
C1—C2	1.393 (6)	C17A—H17A	0.9300
C1—C6	1.383 (7)	C17A—O11A	1.36 (2)
			
O1—Cd1—O2	53.12 (15)	Na2—O10—H10B	109.5
O1—Cd1—O3^i^	131.59 (15)	H10A—O10—H10B	109.2
O1—Cd1—O4^i^	80.31 (12)	C2—C1—C7	121.2 (4)
O1—Cd1—O7^ii^	125.91 (12)	C6—C1—C2	118.9 (4)
O1—Cd1—O8^ii^	92.04 (13)	C6—C1—C7	120.0 (4)
O3^i^—Cd1—O2	173.00 (16)	C1—C2—H2	119.6
O4^i^—Cd1—O2	132.60 (15)	C3—C2—C1	120.7 (4)
O4^i^—Cd1—O3^i^	53.37 (13)	C3—C2—H2	119.6
O4^i^—Cd1—O7^ii^	122.40 (12)	C2—C3—C4	119.7 (4)
O4^i^—Cd1—O8^ii^	78.81 (13)	C2—C3—C8	121.1 (4)
O5—Cd1—O1	125.67 (14)	C4—C3—C8	119.2 (3)
O5—Cd1—O2	90.36 (16)	C3—C4—H4	119.9
O5—Cd1—O3^i^	82.65 (15)	C5—C4—C3	120.2 (4)
O5—Cd1—O4^i^	128.83 (14)	C5—C4—H4	119.9
O5—Cd1—O7^ii^	80.41 (12)	C4—C5—H5	120.1
O5—Cd1—O8^ii^	133.24 (16)	C4—C5—C6	119.8 (5)
O7^ii^—Cd1—O2	85.03 (15)	C6—C5—H5	120.1
O7^ii^—Cd1—O3^i^	94.01 (14)	C1—C6—C5	120.8 (4)
O7^ii^—Cd1—O8^ii^	53.55 (14)	C1—C6—H6	119.6
O8^ii^—Cd1—O2	93.07 (11)	C5—C6—H6	119.6
O8^ii^—Cd1—O3^i^	91.92 (10)	O1—C7—C1	118.1 (5)
O1—Na1—Na2^iii^	77.99 (11)	O2—C7—O1	121.4 (4)
O1—Na1—O4^i^	77.84 (17)	O2—C7—C1	120.5 (4)
O1—Na1—O11B	104.1 (3)	O3—C8—O4	121.4 (4)
O1—Na1—O11A	97.1 (6)	O3—C8—C3	119.9 (4)
O3^iv^—Na1—Na2^iii^	77.96 (14)	O4—C8—C3	118.7 (4)
O3^iv^—Na1—O1	151.1 (2)	C10—C9—C14	119.7 (4)
O3^iv^—Na1—O4^i^	94.59 (15)	C10—C9—C15	119.8 (4)
O3^iv^—Na1—O11B	82.9 (3)	C14—C9—C15	120.5 (4)
O3^iv^—Na1—O11A	93.0 (6)	C9—C10—H10	120.0
O4^i^—Na1—Na2^iii^	41.86 (11)	C9—C10—C11	120.0 (4)
O4^i^—Na1—O11B	177.4 (3)	C11—C10—H10	120.0
O4^i^—Na1—O11A	171.5 (7)	C10—C11—C16	119.6 (4)
O9—Na1—Na2^iii^	130.1 (2)	C12—C11—C10	119.9 (4)
O9—Na1—O1	95.8 (2)	C12—C11—C16	120.4 (4)
O9—Na1—O3^iv^	112.0 (2)	C11—C12—H12	120.0
O9—Na1—O4^i^	88.3 (2)	C13—C12—C11	120.1 (4)
O9—Na1—O11B	93.2 (4)	C13—C12—H12	120.0
O9—Na1—O11A	85.4 (8)	C12—C13—H13	119.7
O11B—Na1—Na2^iii^	136.6 (4)	C12—C13—C14	120.5 (4)
O11A—Na1—Na2^iii^	144.3 (8)	C14—C13—H13	119.7
Cd1^v^—Na2—Na1^v^	55.94 (8)	C9—C14—H14	120.1
O4^vi^—Na2—Cd1^v^	38.59 (8)	C13—C14—C9	119.9 (4)
O4^vi^—Na2—Na1^v^	37.86 (11)	C13—C14—H14	120.1
O5—Na2—Cd1^v^	102.07 (15)	O5—C15—C9	117.2 (4)
O5—Na2—Na1^v^	57.70 (14)	O6—C15—O5	121.7 (4)
O5—Na2—O4^vi^	95.45 (19)	O6—C15—C9	121.1 (4)
O5—Na2—O7^ii^	83.03 (18)	O7—C16—C11	118.4 (4)
O5—Na2—O10	104.5 (2)	O8—C16—O7	122.1 (4)
O7^ii^—Na2—Cd1^v^	133.31 (13)	O8—C16—C11	119.5 (3)
O7^ii^—Na2—Na1^v^	92.36 (13)	C17B—O11B—Na1	112.8 (9)
O7^ii^—Na2—O4^vi^	94.98 (14)	C17B—N1—C18B	120.6 (10)
O7^ii^—Na2—O10	80.5 (2)	C17B—N1—C19B	127.4 (12)
O8^vii^—Na2—Cd1^v^	38.84 (9)	C19B—N1—C18B	111.8 (9)
O8^vii^—Na2—Na1^v^	89.00 (13)	C19A—N1—C18A	106.0 (13)
O8^vii^—Na2—O4^vi^	77.02 (13)	C17A—N1—C18A	116.8 (12)
O8^vii^—Na2—O5	110.18 (16)	C17A—N1—C19A	136.9 (17)
O8^vii^—Na2—O7^ii^	164.93 (18)	O11B—C17B—H17B	119.1
O8^vii^—Na2—O10	102.3 (2)	N1—C17B—O11B	121.8 (13)
O10—Na2—Cd1^v^	139.3 (2)	N1—C17B—H17B	119.1
O10—Na2—Na1^v^	161.7 (2)	N1—C18B—H18A	109.5
O10—Na2—O4^vi^	158.8 (2)	N1—C18B—H18B	109.5
Cd1—O1—Na1	101.49 (13)	N1—C18B—H18C	109.5
C7—O1—Cd1	98.4 (3)	H18A—C18B—H18B	109.5
C7—O1—Na1	159.7 (3)	H18A—C18B—H18C	109.5
C7—O2—Cd1	87.1 (3)	H18B—C18B—H18C	109.5
Na1^vii^—O3—Cd1^viii^	117.93 (19)	N1—C19B—H19A	109.5
C8—O3—Cd1^viii^	90.1 (3)	N1—C19B—H19B	109.5
C8—O3—Na1^vii^	143.6 (3)	N1—C19B—H19C	109.5
Cd1^viii^—O4—Na1^viii^	97.02 (13)	H19A—C19B—H19B	109.5
Cd1^viii^—O4—Na2^ix^	97.42 (13)	H19A—C19B—H19C	109.5
Na1^viii^—O4—Na2^ix^	100.28 (18)	H19B—C19B—H19C	109.5
C8—O4—Cd1^viii^	94.9 (3)	N1—C18A—H18D	109.5
C8—O4—Na1^viii^	146.5 (3)	N1—C18A—H18E	109.5
C8—O4—Na2^ix^	109.1 (3)	N1—C18A—H18F	109.5
Na2—O5—Cd1	99.83 (14)	H18D—C18A—H18E	109.5
C15—O5—Cd1	102.4 (3)	H18D—C18A—H18F	109.5
C15—O5—Na2	157.7 (3)	H18E—C18A—H18F	109.5
Na2^x^—O7—Cd1^x^	96.46 (14)	N1—C19A—H19D	109.5
C16—O7—Cd1^x^	93.8 (3)	N1—C19A—H19E	109.5
C16—O7—Na2^x^	164.5 (3)	N1—C19A—H19F	109.5
Na2^iv^—O8—Cd1^x^	105.91 (16)	H19D—C19A—H19E	109.5
C16—O8—Cd1^x^	90.5 (2)	H19D—C19A—H19F	109.5
C16—O8—Na2^iv^	149.8 (3)	H19E—C19A—H19F	109.5
Na1—O9—H9A	110.8	N1—C17A—H17A	123.6
Na1—O9—H9B	112.4	N1—C17A—O11A	113 (2)
H9A—O9—H9B	106.7	O11A—C17A—H17A	123.6
Na2—O10—H10A	109.8	C17A—O11A—Na1	136.8 (17)
			
Cd1—O1—C7—O2	1.6 (5)	C4—C3—C8—O3	−164.6 (4)
Cd1—O1—C7—C1	−179.3 (3)	C4—C3—C8—O4	15.9 (6)
Cd1—O2—C7—O1	−1.5 (4)	C4—C5—C6—C1	0.0 (7)
Cd1—O2—C7—C1	179.5 (4)	C6—C1—C2—C3	−0.5 (6)
Cd1^viii^—O3—C8—O4	−5.0 (4)	C6—C1—C7—O1	−1.3 (6)
Cd1^viii^—O3—C8—C3	175.5 (3)	C6—C1—C7—O2	177.8 (5)
Cd1^viii^—O4—C8—O3	5.2 (4)	C7—C1—C2—C3	180.0 (4)
Cd1^viii^—O4—C8—C3	−175.2 (3)	C7—C1—C6—C5	179.6 (4)
Cd1—O5—C15—O6	−6.1 (6)	C8—C3—C4—C5	179.5 (4)
Cd1—O5—C15—C9	173.6 (3)	C9—C10—C11—C12	−1.4 (6)
Cd1^x^—O7—C16—O8	1.9 (4)	C9—C10—C11—C16	175.0 (4)
Cd1^x^—O7—C16—C11	−178.2 (3)	C10—C9—C14—C13	0.6 (7)
Cd1^x^—O8—C16—O7	−1.9 (4)	C10—C9—C15—O5	179.3 (4)
Cd1^x^—O8—C16—C11	178.3 (3)	C10—C9—C15—O6	−1.0 (7)
Na1—O1—C7—O2	−166.8 (7)	C10—C11—C12—C13	0.5 (7)
Na1—O1—C7—C1	12.2 (11)	C10—C11—C16—O7	177.1 (4)
Na1^vii^—O3—C8—O4	−147.3 (4)	C10—C11—C16—O8	−3.1 (6)
Na1^vii^—O3—C8—C3	33.1 (7)	C11—C12—C13—C14	1.0 (7)
Na1^viii^—O4—C8—O3	115.7 (5)	C12—C11—C16—O7	−6.5 (6)
Na1^viii^—O4—C8—C3	−64.7 (7)	C12—C11—C16—O8	173.4 (4)
Na1—O11B—C17B—N1	148.4 (9)	C12—C13—C14—C9	−1.5 (7)
Na2^ix^—O4—C8—O3	−94.4 (4)	C14—C9—C10—C11	0.8 (6)
Na2^ix^—O4—C8—C3	85.2 (4)	C14—C9—C15—O5	1.0 (6)
Na2—O5—C15—O6	−179.4 (8)	C14—C9—C15—O6	−179.2 (5)
Na2—O5—C15—C9	0.4 (13)	C15—C9—C10—C11	−177.4 (4)
Na2^x^—O7—C16—O8	−129.6 (10)	C15—C9—C14—C13	178.9 (4)
Na2^x^—O7—C16—C11	50.2 (13)	C16—C11—C12—C13	−175.9 (4)
Na2^iv^—O8—C16—O7	122.2 (5)	N1—C17A—O11A—Na1	−116 (3)
Na2^iv^—O8—C16—C11	−57.7 (7)	C17B—N1—C17A—O11A	6.5 (16)
C1—C2—C3—C4	0.9 (6)	C18B—N1—C17B—O11B	−0.2 (17)
C1—C2—C3—C8	−179.5 (4)	C18B—N1—C17A—O11A	−174 (2)
C2—C1—C6—C5	0.0 (7)	C19B—N1—C17B—O11B	−173.3 (11)
C2—C1—C7—O1	178.3 (4)	C18A—N1—C17B—O11B	175.3 (18)
C2—C1—C7—O2	−2.6 (6)	C18A—N1—C17A—O11A	2 (3)
C2—C3—C4—C5	−0.9 (7)	C19A—N1—C17B—O11B	−142 (6)
C2—C3—C8—O3	15.8 (6)	C19A—N1—C17A—O11A	174 (2)
C2—C3—C8—O4	−163.7 (4)	C17A—N1—C17B—O11B	0.1 (14)
C3—C4—C5—C6	0.5 (7)		

Symmetry codes: (i) *x*, *y*−1, *z*; (ii) *x*−1, *y*, *z*; (iii) −*y*, *x*, *z*−1/4; (iv) −*y*+1, *x*, *z*−1/4; (v) *y*, −*x*, *z*+1/4; (vi) *y*−1, −*x*, *z*+1/4; (vii) *y*, −*x*+1, *z*+1/4; (viii) *x*, *y*+1, *z*; (ix) −*y*, *x*+1, *z*−1/4; (x) *x*+1, *y*, *z*.

### Hydrogen-bond geometry (Å, º)

**Table:**

*D*—H···*A*	*D*—H	H···*A*	*D*···*A*	*D*—H···*A*
O9—H9*B*···O6	0.90	2.22	3.074 (8)	159
O10—H10*B*···O6^vii^	0.86	2.29	3.073 (8)	152
C4—H4···O3^xi^	0.93	2.49	3.385 (6)	161
C6—H6···O1	0.93	2.48	2.791 (5)	100
C6—H6···O11*B*	0.93	2.48	3.347 (14)	155
C10—H10···O10^iv^	0.93	2.59	3.277 (8)	131
C14—H14···O8^vii^	0.93	2.48	3.366 (5)	159
C18*B*—H18*A*···*Cg*3^iv^	-	2.54	3.387 (11)	148
C19*B*—H19*B*···*Cg*4^xii^	-	2.93	3.696 (15)	138
C19*A*—H19*D*···*Cg*4^xii^	-	2.67	3.53 (4)	151

Symmetry codes: (iv) −*y*+1, *x*, *z*−1/4; (vii) *y*, −*x*+1, *z*+1/4; (xi) −*y*+1, *x*+1, *z*−1/4; (xii) −*x*+1, −*y*+1, *z*−1/2.
